# Identification and immunological characterization of cuproptosis-related molecular clusters in idiopathic pulmonary fibrosis disease

**DOI:** 10.3389/fimmu.2023.1171445

**Published:** 2023-05-17

**Authors:** Xuefeng Shi, Zhilei Pan, Weixiu Cai, Yuhao Zhang, Jie Duo, Ruitian Liu, Ting Cai

**Affiliations:** ^1^ Department of Experimental Medical Science, Ningbo No.2 Hospital, Ningbo, China; ^2^ Department of Pulmonary and Critial Care medicine, Qinghai provincial people’s hospital, Xining, China; ^3^ State Key Laboratory of Biochemical Engineering, Institute of Process Engineering, Chinese Academy of Sciences, Beijing, China; ^4^ Cancer Center, Department of Neurosurgery, Zhejiang Provincial People’s Hospital, Affiliated People’s Hospital, Hangzhou Medical College, Hangzhou, China

**Keywords:** idiopathic pulmonary fibrosis disease, cuproptosis, machine learning, immune infiltration, molecular clusters

## Abstract

**Background:**

Idiopathic pulmonary fibrosis (IPF) has attracted considerable attention worldwide and is challenging to diagnose. Cuproptosis is a new form of cell death that seems to be associated with various diseases. However, whether cuproptosis-related genes (CRGs) play a role in regulating IPF disease is unknown. This study aims to analyze the effect of CRGs on the progression of IPF and identify possible biomarkers.

**Methods:**

Based on the GSE38958 dataset, we systematically evaluated the differentially expressed CRGs and immune characteristics of IPF disease. We then explored the cuproptosis-related molecular clusters, the related immune cell infiltration, and the biological characteristics analysis. Subsequently, a weighted gene co-expression network analysis (WGCNA) was performed to identify cluster-specific differentially expressed genes. Lastly, the eXtreme Gradient Boosting (XGB) machine-learning model was chosen for the analysis of prediction and external datasets validated the predictive efficiency.

**Results:**

Nine differentially expressed CRGs were identified between healthy and IPF patients. IPF patients showed higher monocytes and monophages M0 infiltration and lower naive B cells and memory resting T CD4 cells infiltration than healthy individuals. A positive relationship was found between activated dendritic cells and CRGs of LIPT1, LIAS, GLS, and DBT. We also identified cuproptosis subtypes in IPF patients. Go and KEGG pathways analysis demonstrated that cluster-specific differentially expressed genes in Cluster 2 were closely related to monocyte aggregation, ubiquitin ligase complex, and ubiquitin-mediated proteolysis, among others. We also constructed an XGB machine model to diagnose IPF, presenting the best performance with a relatively lower residual and higher area under the curve (AUC= 0.700) and validated by external validation datasets (GSE33566, AUC = 0.700). The analysis of the nomogram model demonstrated that XKR6, MLLT3, CD40LG, and HK3 might be used to diagnose IPF disease. Further analysis revealed that CD40LG was significantly associated with IPF.

**Conclusion:**

Our study systematically illustrated the complicated relationship between cuproptosis and IPF disease, and constructed an effective model for the diagnosis of IPF disease patients.

## 1 Introduction

IPF is among the most severe form of interstitial pneumonia, characterized by chronic and progressive lung scars and usual interstitial pneumonia (1). IPF has a poor prognosis, with a median life expectancy of only 2-3 years from diagnosis (2). Epidemiological studies of North America, the US, and Europe demonstrated that the number of IPF patients increased, placing a growing economic burden on global health care (1). Currently, the primary drugs used to treat IPF are pirfenidone and nidanib. Nevertheless, there are some limitations in preventing disease progression and improving the quality of life of patients because of the treatment efficacy of Individual differences, and side effects (gastrointestinal intolerance, skin reactions and diarrhea) caused by the Nintedanib and Prefenidone (3). IPF is the result of various mechanisms. Alveolar epithelial injury and infiltration of inflammatory cells, such as neutrophils, macrophages, and lymphocytes, are the primary causes of the destruction of lung tissue structure, alveolar atrophy and collapse, and regression of pulmonary vessels (4). The accumulation of extracellular matrix in lung tissue leads to fibroblast foci and collagen fiber reconstruction (5). In addition, the development of IPF is favored by the interaction of epithelial-mesenchymal transition (EMT), interleukin, TGF-β, and oxidative stress.

Cuproptosis, a novel unique non-apoptotic programmed cell death, targets and leads the aggregation of fatty acylated components and the loss of Fe-S cluster-containing proteins, causing proteotoxic stress and cell death (6). At present, more articles reveled the cuproptosis-related genes (CRGs) as a bio-marker play an important role in the development of disease, such as stomach adenocarcinoma (STAD), hepatocellular carcinoma (HCC) and head and neck squamous carcinoma (HNSC) (7–9). Furthermore, copper is essential for all living organisms and serves as a catalyst, antioxidant defense, autophagy, and even arouses immune activation (10). Notably, copper homeostasis strongly correlates with the concentration of T cells, neutrophils, and macrophages (11). In the development of pulmonary fibrosis, H2O2 was increased in alveolar macrophages due to the translocation of Cu and Zn-SOD to the mitochondrial intermembrane space (12). In addition, NLRP3, a cuproptosis gene, was involved in TGF-β and EMT signaling pathways and promoted fibrosis progression (13, 14). Therefore, we hypothesize that cuproptosis-related genes (CRGs) may play a role in developing IPF. This study investigated the underlying mechanism and immune cell infiltration on IPF and analyzed the effect of CRGs on IPF. In this study, the underlying mechanism and immune cell infiltration of IPF was investigated, and the effect of CRGs on IPF was analyzed.

## 2 Materials and methods

### 2.1 Raw data acquisition and processing

Three datasets (GSE38958, GSE28042, and GSE33566) were downloaded from the database of the website GEO (GEO, www.ncbi.nlm.nih.gov/geo). Database GSE38958 (platform GPL5175), which includes 45 healthy and 70 IPF blood samples, was selected to analyze the relationship between CRGs and IPF and construct the machine learning model to diagnose IPF. Datasets GSE28042 (GPL6480 platform) (containing 19 healthy and 75 IPF blood samples) and GSE33566 (GPL6480) (containing 30 healthy and 93 IPF blood samples) were used for the validation of the IPF prediction model and following analysis. The three datasets were processed with limma package and normalized using the normalizeBetweenArrays method.

### 2.2 CRGs difference expression and correlation analysis

According to Peter Tsvetkov’s report (6), 19 cuprotosis-related genes were reported and analyzed, including NFE2L2, NLRP3, ATP7B, ATP7A, SLC31A1, FDX1, LIAS, LIPT1, LIPT2, DLD, DLAT, PDHA1, PDHB, MTF1, GLS, CDKN2A, DBT, GCSH, and DLST. These genes were selected for analysis of CRGs expression in the blood of 45 healthy and 70 IPF patients. The differentially expressed cuprotosis-related genes was analyzed by the wilcox.test, and *p*-values < 0.05 was considered to be significantly different. The heatmap and boxplot were exhibited using R packages heatmap and ggpubr. Then, the conspicuous expression of CRGs in IPF was selected for correlation analysis. The results were exhibited using the R packages corrplot (version 0.92) and circlize (15). *P*-values below 0.05 represented a significant correlation.

### 2.3 Relationship between cuproptosis-related genes expression and immunity

CIBERSORT R package (16) and LM22 signature matrix were applied to esimate the relative abundance of 22 types of immune cells infiltrated in IPF patients. Correlations between CRGs and immune cells infiltration level in IPF were performed using the R packages tidyverse (17), ggplot2 (18), and reshape2. The sum of the 22 immune cells proportions in each sample was 1 (16), and *p* < 0.05 represented a significant correlation.

### 2.4 IPF patients classification analysis

The R package ConsensusClusterPlus (19) and the k-means algorithm with 1,000 iterations were applied to classify 70 IPF samples into different clusters based on the differentially expressed CRGs profile acquired from 2.2. The maximum subtype k was 9 and the optimal clusters numeber was comprehensively evaluated based on the result of the cumulative distribution function (CDF) curve, consensus matrix and consistent cluster score (> 0.9).

### 2.5 Gene set variation analysis

GSVA, a non-parametric unsupervised analytical method, is mainly used to evaluate the results of gene enrichment by R packages limma, GSEABase, and GSVA. We downloaded “c2.cp.kegg.v7.4.symbols” and “c5.go.bp.v7.5.1.symbols” from the MSigDB website database. Finally, the top 10 GO and KEGG pathways were selected for statistical analysis and ridge mapping. The absolute value of t value of GSVA score more than 2 was considered as significantly altered.

### 2.6 Weighted gene co-expression network analysis analysis

Co-expression modules were identified by the R package WGCNA (20). The top 25% of genes with the highest variance were used for subsequent WGCNA analysis. We then constructed an adjacency matrix with the optimal soft power value and converted it into a topological overlap matrix (TOM). Based on the hierarchical clustering tree algorithm, the modules were determined using the TOM dissimilarity measure (1-TOM) and the minimum module size was set to 100. Each module was assigned a random color. Module eigengene represented the gene expression profiles in one module. The correlation between genes, clinical phenotype, modules, and disease status were also identified. The modular significance showed the relationship between modules and disease status. Gene significance was described as the correlation between a gene with the clinical phenotype.

### 2.7 Construction and verification of multiple machine learning model

Four machine-learning models: Support Vector Machines (SVM), XGB, generalized linear model (GLM), and Random Forest (RF) models were built by the R package caret, and all the models worked with default parameters and assessed *via* 5-fold cross-validation. Data were randomly divided into a training set (70%, N=81) and a test set (30%, N=35). Interpretive analysis of the 4 models was performed by the DALEX package (21), and then the cumulative residual distribution map and boxplot distribution map of these machine-learning models were visualized. The ROC curves were obtained and visualized using the pROC R package. Next, the optimal learning model was determined, and the top 4 key genes were selected as the predictive genes related to the IPF. Subsequently, the ability of the predictive model was validated with GSE33566 using the ROC analysis. In addition, we performed the correlation between four key genes and TGF-β and constructed a gene-gene interaction network by the GeneMANIA website for key genes (http://www.genemania.org). R package rms was used to build a nomogram model, and the predictive power of the nomogram model was tested by the calibration curve and decision curve analysis (DCA).

### 2.8 The analysis of clinical features

To determine the relationship between key genes and clinical indicators associated with IPF, including age, diffusing capacity of the lung for carbon monoxide (DLCO), and FVC, the spearman correlation analysis was performed to explore the correlations. R packages ggplot2, ggpubr (version 0.4.0), and ggExtra (version 0.10.0) were used to draw the scatter plot. *P* < 0.05 represented a significant correlation and R represented a correlation coefficient.

## 3 Results

### 3.1 CRGs expression and immune activation in IPF

**Figure 1:**
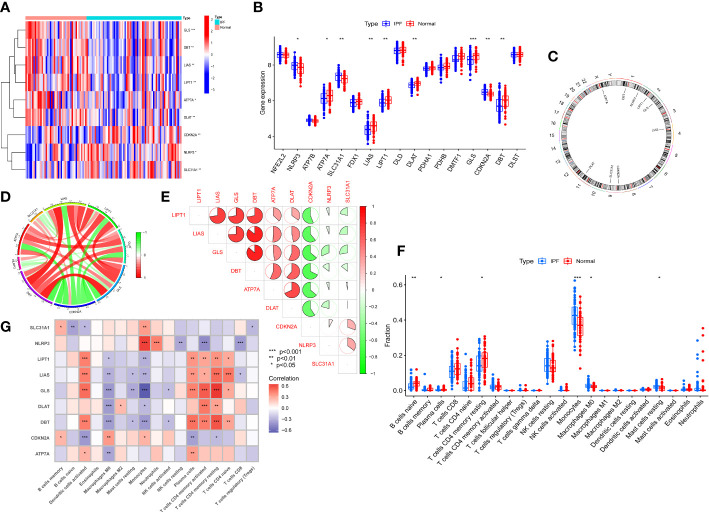
CRGs expression and immune cells infiltration in IPF. **(A)** Significantly differential expressed CRGs between normal individuals and IPF patients -Heatmap. **(B)** The CRGs expression between Normal group and IPF group. **(C)** The location of 9 CRGs on chromosomes. **(D)** Correlation of differentially expressed CRGs - Cyclograph. **(E)** Correlation of differentially expressed CRGs, red and green represent positive correlation and negative correlation, respectively-Pie chart. **(F)** The relative percent of immune cells in Normal and IPF groups. **(G)** The differentially expressed CRGs expression in immune cells. *p< 0.05, **p< 0.01, ***p< 0.001.

We systematically analyzed the differentially expressed curproptosis genes between healthy and IPF patients using the GSE38958 database. There were 9 CRGs with significant differences in IPF patients including, NLRP3, ATP7B, ATP7A, SLC31A1, FDX1, LIAS, LIPT1, DLAT, GLS, CDKN2A, and DBT. Among them, 3 CRGs in IPF samples were higher than that in healthy subjects, including NLRP3, SLC31A1, and CDKN2A, while others exhibited a lower expression, especially GLS (**Figures 1A, B**). The location of 9 CRGs on chromosomes is shown in **Figure 1C**. We also performed the correlation analysis among the 9 CRGs to examine whether these genes play an essential functional role in the progression of IPF. The results showed an apparent synergistic effect among the LIPT1, LIAS, GLS, DBT, ATP7A, and DLAT, and the most robust antagonistic effect was found between CDKN2A and LIPT1, LIAS, GLS, DBT, ATP7A and DLAT (**Figure 1E**). The Cyclograph was constructed to detect further the relationships of the differentially expressed CRGs (**Figure 1D**).

We estimated the relative percent of 22 types of immune cells in healthy and IPF patients to find immune cell infiltration differences. The boxplot results revealed that IPF patients had higher immune cell infiltration of Monocytes and Monophages M0 than healthy subjects but lower naive B cells and memory resting T cells CD4 infiltration (**Figure 1F**). Meanwhile, we also examined the correlation between CRGs and immune infiltration. The results showed a strong positive relationship between activated dendritic cells and LIPT1, LIAS, GLS, and DBT. In addition, these four genes also showed a positive relationship with plasma cells, memory activated and resting T Cells CD4, and naive T cells CD4. However, a negative relationship was found between the macrophages M0 and LIPT1, LIAS, GLS, DLAT, DBT, and ATP7A. The monocytes displayed the most robust positive relationship with NLRP3 and a negative relationship with GLS (**Figure 1G**).

### 3.2 Identification of cuproptosis related IPF subtypes

**Figure 2:**
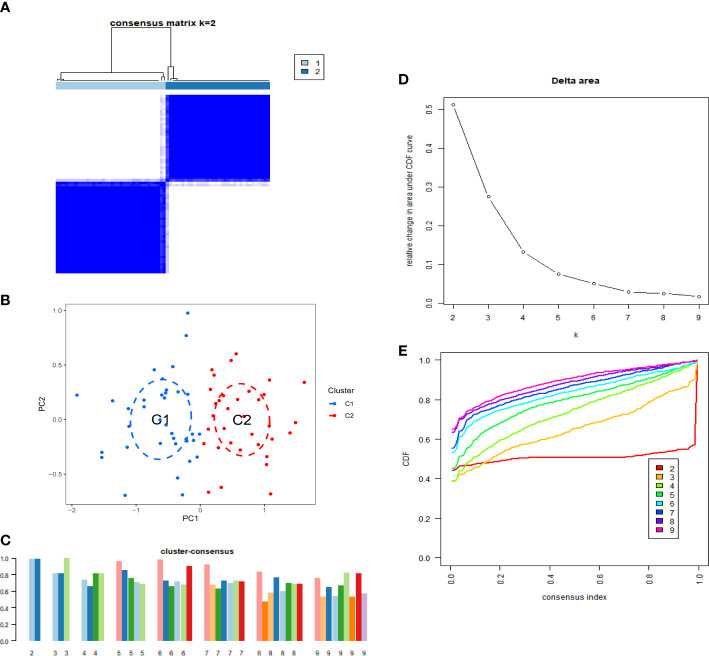
Identification of cuproptosis-related IPF subtype. **(A)** Consensus matrix when k=2. **(B)** CDF delta area curves when k was ranged 2 to 9. **(C)** Representative cumulative distribution function (CDF) curves. **(D)** The score of consensus clustering. **(E)** Principal component analysis (PCA) of two subtypes.

To elucidate the cuproptosis-related expression patterns in IPF, we classified 70 IPF samples based on differentially expressed CRGs. The cluster numbers were most stable when the k value was set to two (k = 2). Moreover, the CDF curves fluctuated within a minimum range at a consensus index of 0.2 to 0.8 (**Figures 2A, E**). When k = 2 to 9, the area under the CDF curves exhibited the difference between the two CDF curves (k and k-1) (**Figure 2D**). Furthermore, the consistency score of each subtype was >0.9 only when k = 2. (**Figure 2C**). Furthermore, the two clusters showed significant differences (**Figure 2B**).

### 3.3 CRGs and immune cell infiltration in different cuproptosis related IPF subtypes

**Figure 3:**
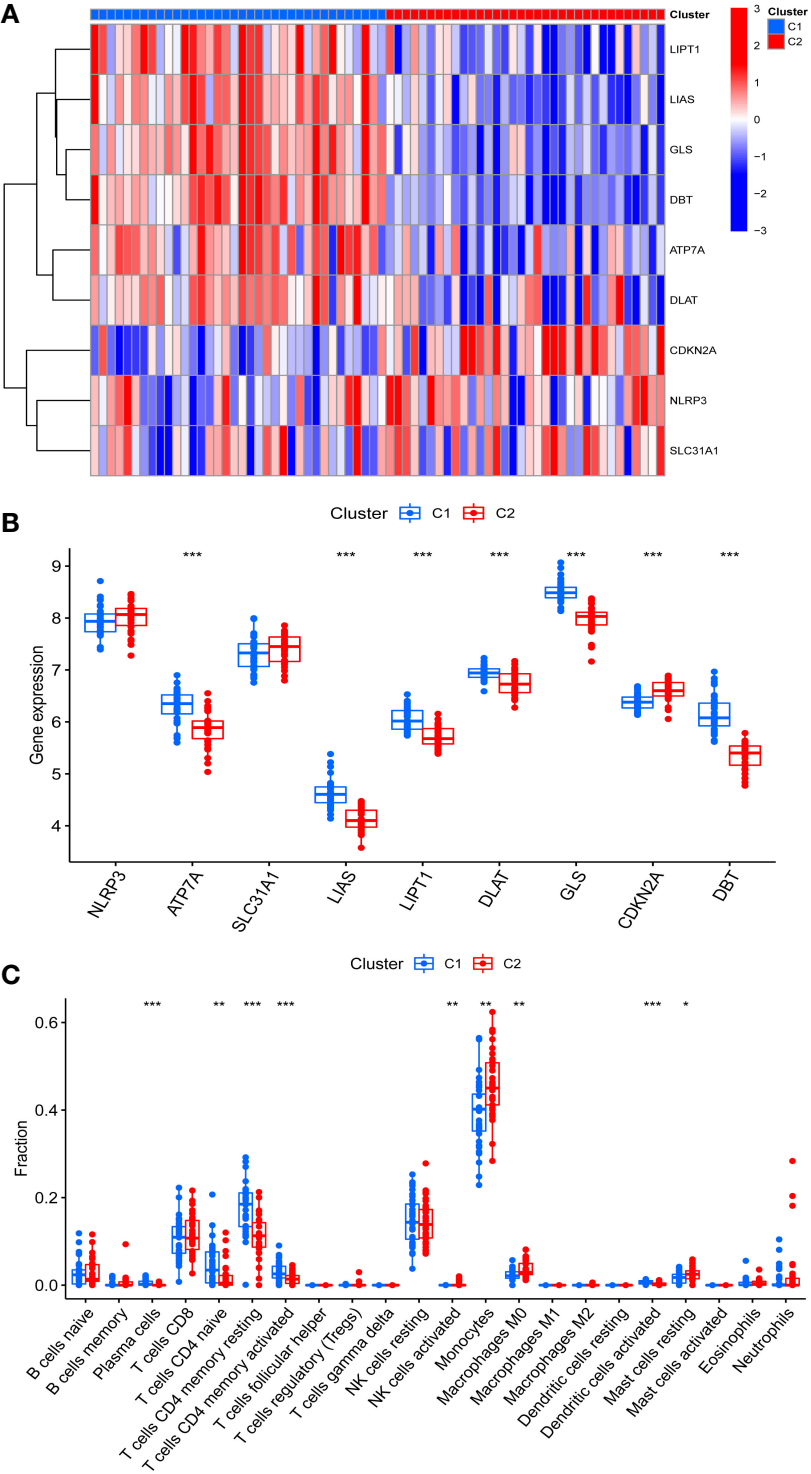
Identification of CRGs expression and immune characteristics between the two cuproptosis related IPF subtype (clusters). **(A)** CRGs expression between the two cuproptosis related IPF clusters - Heatmap. **(B)** CRGs expression between the two cuproptosis related IPF clusters. **(C)** The relative percent of 22 infiltrated immune cells between two cuproptosis related IPF clusters. *p< 0.05, **p< 0.01, ***p< 0.001.

The differences in immune cell infiltration and differentially expressed CRGs were also examined in different cuproptosis-related IPF subgroups, and there were 9 differentially expressed CRGs between Cluster 1 and Cluster 2. ATP7A, LIAS, LIPT1, DLAT, GLS, and DBT overexpressed in Cluster 1, and CDKN2A overexpressed in Cluster 2 (**Figures 3A, B**). Moreover, Cluster 1 exhibited higher immune cell infiltration of naive T cells CD4, memory resting and activated T cells CD4, but a lower level of monocytes, macrophages M0, and resting mast cells (**Figure 3C**).

### 3.4 GSVA analysis

**Figure 4:**
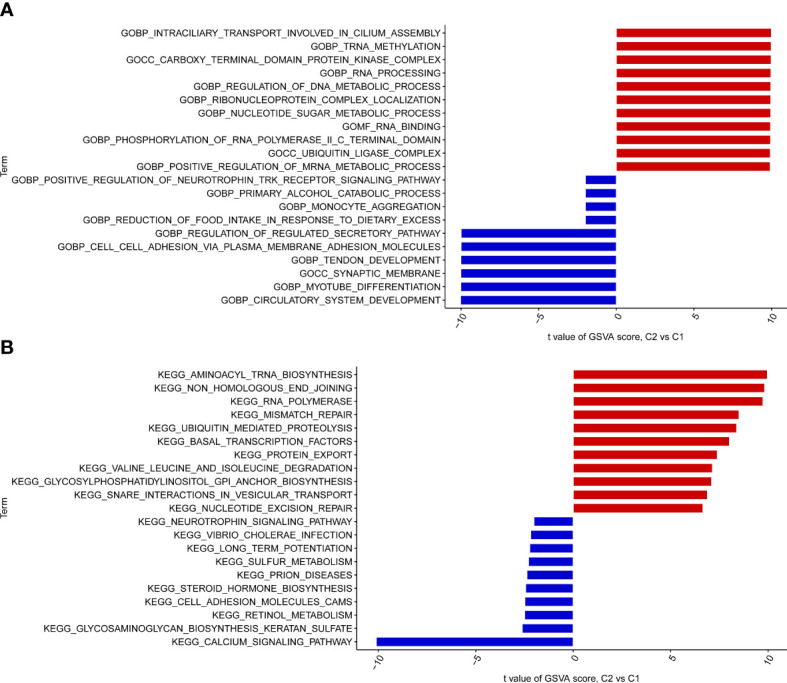
GO enrichment and KEGG pathway enrichment between the two cuproptosis related IPF subtype (clusters). **(A)** GO enrichment. **(B)** KEGG pathway enrichment.

To explore the GO function and KEGG pathway in different clusters, the GSCA algorithm was applied to quantify the test value of GSVA between clusters. The results of GO analysis indicated that Cluster 2 IPF group was enriched in the ubiquitin ligase complex, ubiquitin mediated proteolysis, tRNA methylation, monocyte aggregation, nucleotide sugar metabolic process, cell-cell adhesion *via* plasma membrane adhesion molecules, circulatory system development, myotube differentiation, and synaptic membrane, among others (**Figure 4A**). KEGG pathway enrichment showed that Cluster 2 IPF was enriched in aminoacyl tRNA biosynthesis, RNA polymerase, and calcium signaling pathway, among others (**Figure 4B**).

### 3.5 WGCNA co-expression analysis

**Figure 5:**
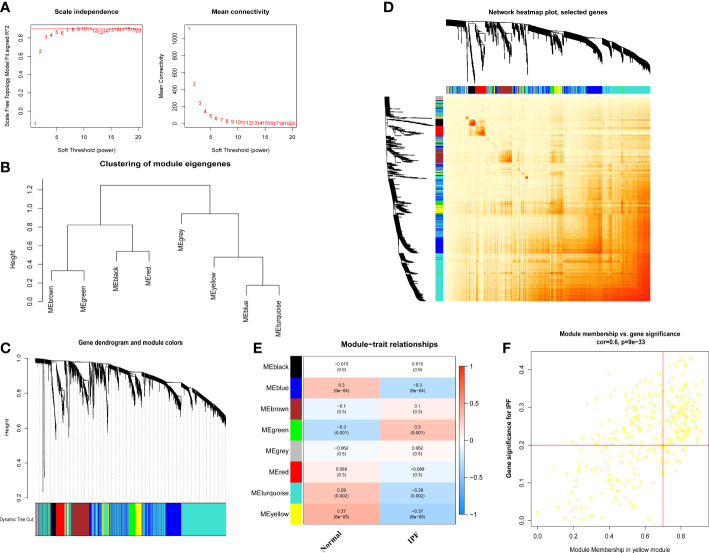
Co-expression network of differential expressed genes between IPF patients and normal individuals. **(A)** Exponential curve fitting and mean connectivity of power value. **(B)** The correlation between different modules in dendrogram. **(C)** Gene clustering dendrogram with dynamic identification of modules. Different colors show distinct co-expression modules. **(D)** Network heatmap of the correlation among 8 modules. **(E)** Module-trait relationships. Each row represents a module; each column represents a clinical status. **(F)** Scatter plot between module membership in yellow module and the gene significance for IPF.

To find out the essential gene modules related to the IPF, the co-expression network and modules were constructed using the WGCNA algorithm, and the top 25% of differently expressed genes were opted to further analysis. When the optimal value of soft power was set to 5, the co-expressed gene modules were identified, and R^2^ was equal to 0.92 (**Figure 5A**). Thus, 8 distinct modules with different colors were obtained, and the topological overlap matrix was displayed (**Figures 5B–D**). The yellow module strongly correlated with the IPF with a correlation coefficient of 0.6 and *p* value of 9×e-24 (**Figure 5E**). A total of 253 genes were in the yellow module, as shown in **Figure 5F**.

**Figure 6:**
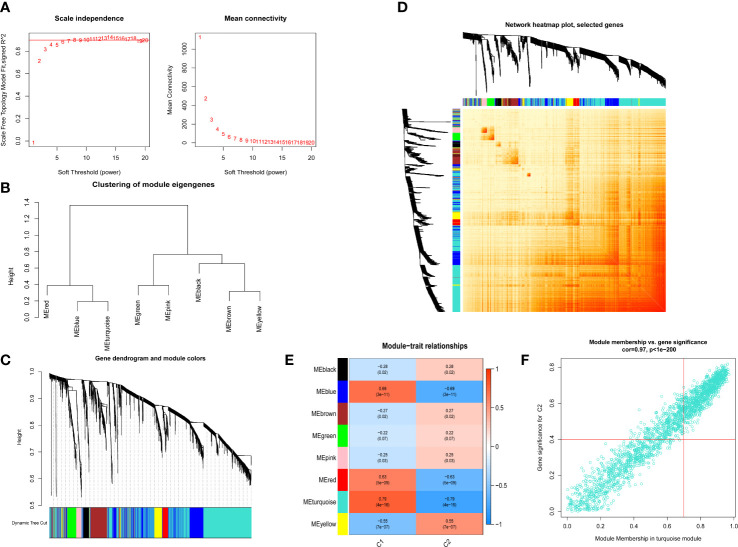
Co-expression network of differential expressed genes between two cuproptosis related IPF clusters. **(A)** Exponential curve fitting of power value. **(B)** The correlation between different modules in dendrogram. **(C)** Gene clustering dendrogram with dynamic identification of modules. Different colors show distinct co-expression modules. **(D)** Network heatmap of the correlation among 8 modules. **(E)** Module-trait relationships. Each row represents a module; each column represents a clinical status. C1 and C2 represent cluster 1 and cluster 2, respectively. **(F)** Scatter plot between module membership in turquoise module and the gene significance for cluster 2.

We also used the R package WGCNA to analyze the correlations between cuproptosis clusters and critical genes modules. The scale-free network was ensured when β = 4 (scare-free R^2 ^= 0.97) (**Figure 6A**). There were 8 significant modules determined (**Figures 6B–D**), and the turquoise module had the highest relationship with IPF (**Figure 6E**). The scatter plot portrayed the relationship between members in the turquoise module and the significant gene of Cluster 2 (**Figure 6F**).

### 3.6 Establishment and evaluation of machine learning

**Figure 7:**
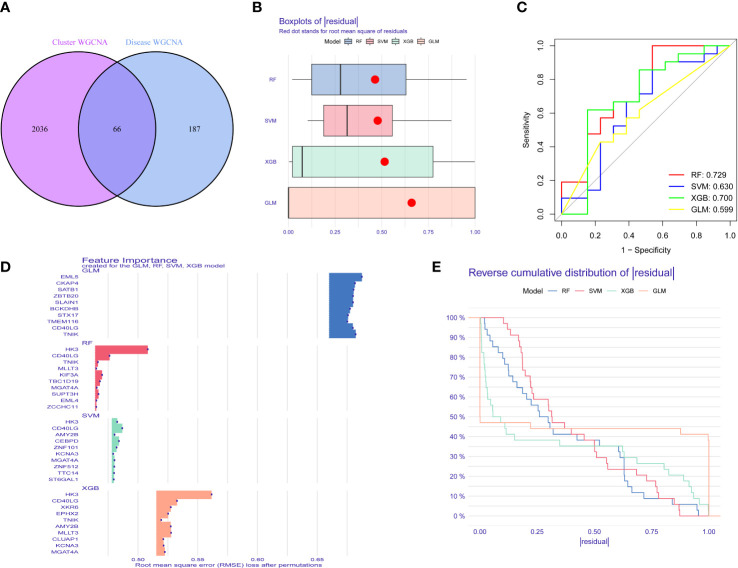
The construction and verification of Study machine learning. **(A)** The core gene of differently expressed gene in IPF and IPF clusters. **(B)** Boxplots of four machine learning models. **(C)** ROC analysis of machine learning models. **(D)** Top gene of four models. **(E)** Cumulative residual distribution of XGB, RF, GLM and SVM machine learning models.

To identify specific genes with a high diagnostic capacity for IPF, 66 core genes (**Figure 7A**) were used to train a machine-learning model with different methods, including SVM, XGB, GLM, and RF. XGB and GLM models displayed a relatively low residual (**Figures 7B, E**). Subsequently, the top 10 feature variables of each method were ranked according to the root mean square error (RMSE, **Figure 7D**). Moreover, all four machine learning models were evaluated for the discriminative performance by calculating receiver operating characteristic (ROC) curves, and all the performance of models were compared by AUC-ROC value (RF, AUC = 0.729; SVM, AUC = 0.630; XGB, AUC= 0.700; GLM, AUC= 0.599, **Figure 7C**). Above all, the XGB model was the best model to distinguish IPF. Moreover, the 4 genes, including XKR6, MLLT3, CD40LG and HK3, were applied as predictor genes for further analysis.

**Figure 8:**
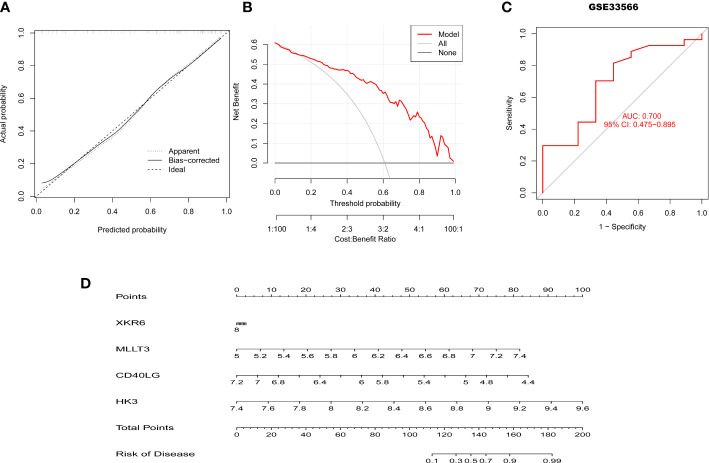
Validation of the 4-gene-based XGB model. **(A, B)** Predictive efficiency of the nomogram model by the DCA **(A)** and calibration curve **(B)**. **(C)** ROC curve of the 4-gene-based XGB model in the GSE33566. **(D)** The construction of nomogram for predicting the rate of IPF based on the 4-gene-based XGB model.

To further assess the predictive efficiency of the XGB model a clinical nomogram was created, which assigns all risk factors to points and judges the IPF risk according to the total points (**Figure 8D**). The R package rms made the calibration curve and DCA to assess the predictive efficiency of the nomogram model. Results showed that the nomogram had high accuracy in diagnosing IPF, with the predicted probability presenting a small error and the decision curve of the model far from the curve of all models (**Figures 8A, B**). We then validated the 4-gene prediction model with ROC analysis, which showed satisfactory performance with an AUC value of 0.7 in the GSE33566 database (healthy vs. IPF patients) (**Figure 8C**). The results indicated that our diagnosis model is effectively distinguishes IPF from healthy patients.

### 3.7 The relationship analysis between clinical characteristics and the 4 critical genes

**Figure 9:**
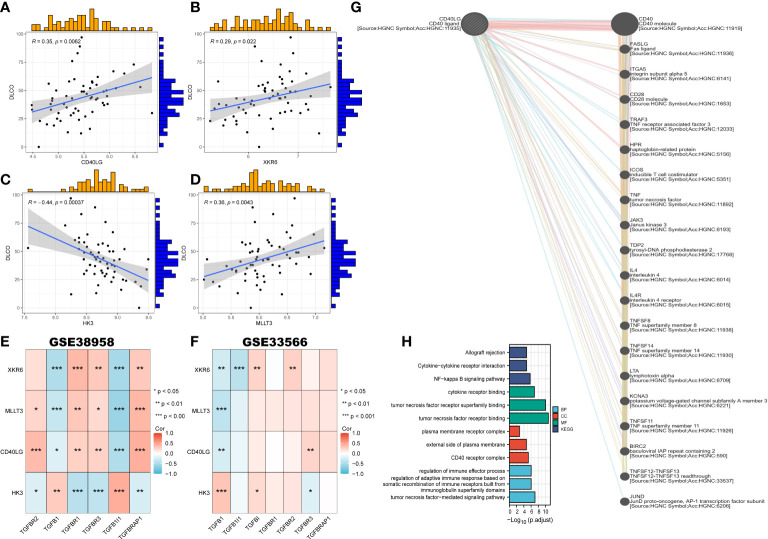
Correlation of clinical characteristics with CRGs based on two datasets and the construction of gene-gene network. **(A–D)** The correlation between key genes and DLCO. **(E, F)** The correlation between four key genes and TGF-β in GSE38958 **(E)** and GSE33566 **(F)**. **(G)** The gene-gene interaction network of CD40LG from GeneMANIA. **(H)** Go enrichment and KEGG pathway enrichment for genes related to CD40LG.

To explore the correlation between clinical characteristics and the 4 most critical genes, we enrolled them in the GSE38958 databases to validate the correlation between the predictor genes and clinical characteristics. DLCO was selected as the factor related to IPF. The results revealed that 3 genes exhibited a positive correlation with DLCO (*p* < 0.05, CD40LG, R = 0.35; XKR6, R = 0.29; MLLT3, R = 0.36), except HK3 (R = -0.44, *p* < 0.01) (**Figures 9A–D**).

We also constructed the heatmap portraying the correlation between the 4 genes and genes related to TGF-β in the GSE38958 and GSE33566 databases. Two databases showed that XKR6, MLLT3, and CD40LG had a negative correlation with TGFβ1, while HK3 presented a positive relationship (**Figure 9E, F**). Meanwhile, the gene-gene interaction network for CD40LG was constructed using GeneMANIA, and the functions with high significance were selected to display (**Figure 9G**). Moreover, the function and pathways analysis revealed that CD40LG was prominently enriched in tumor necrosis factor (TNF) receptor binding, TNF-mediated signaling pathway, CD40 receptor complex, NF-κB signaling pathway, and cytokine and regulation of immune effector process (**Figure 9H**).

## 4 Discussion

IPF is a progressive and irreversible lung disease with different etiology. There is no effective treatment but lung transplantation for IPF patients (22). A new mechanism, copper-dependent cell death, has been reported to be strongly associated with disease progression through the aggregation of lipoylated mitochondrial enzymes and loss of iron-suffer cluster proteins (6). As there was no study about the role of CRGs in IPF patients blood, more studies needed to analysis the relationship between CRGs and IPF in blood samples, and the correlation between CRGs and immune cells in IPF patients. Therefore, we sought to clarify the role of CRGs in the progression of IPF and the effect on the immune microenvironment of IPF patients, which may provide a novel treatment approach for IPF. Additionally, gene signatures related to cuproptosis were used to predict IPF subtypes, and define biomarkers for the diagnosis of IPF.

It’s reported that the CRGs, such as FDX1, LIAS, DLD, PDHA1, PDHB, DLAT, and LIPT1, were down-regulated in the lung tissues of pulmonary fibrosis mouse model, and the same results were obtained via analysis of lung tissues scRNA-seq data for human pulmonary fibrosis (23). In our study, differential expression analysis showed that there were 9 different expressed CRGs in blood samples of IPF patients compared with healthy individuals, suggesting that CRGs may participate in the development of IPF. Of the 9 CRGs, NLRP3, SLC31A1, and CDKN2A were upregulated in IPF, while ATP7A, LIAS, LIPT1, DLAT, GLS, and DBT were downregulated in IPF patients than healthy subjects. It also has been reported that the overactivation of NLRP3 in IPF patients leads to the increased production of Class I of collagens (24, 25), and NLRP3 inflammasome can promote fibrosis *via* pathways involving TGF-β1 and EMT (26). Besides, CDKN2A, a cell cycle negative regulator, is involved in the progression of dysregulated epithelial cell senescence and triggering the activation of fibroblasts and myofibroblasts in IPF patients (27, 28).Therefore, CRGs may attend to the progression of IPF, but more studies are needed.

Subsequently, we further calculated the correlation between the CRGs to clarify the relationship between cuproptosis regulators and IPF. There was an apparent synergistic effect among LIPT1, LIAS, GLS, DBT, ATP7A, and DLAT, and a robust antagonistic effect between CDKN2A and LIPT1, LIAS, GLS, DBT, ATP7A, and DLAT in IPF patients. Moreover, the abundance of immune cells differed between healthy subjects and IPF patients. In this study, IPF patients exhibited high infiltration levels of monocytes, which was consistent with previous studies, and can be considered a biomarker for assessing IPF patients (29). Further, based on the expression landscapes of CRGs, we used unsupervised cluster analysis to illustrate the different cuproptosis regulation patterns in IPF patients. Two distinct cuproptosis-related clusters were identified. We found that most CRGs were downregulated in the Cluster 2 IPF group. In addition, the cluster 2 group had a high infiltration of monocytes and macrophages M0, and low infiltration of naive T cells CD4 and memory resting and activated T cells CD4. Elevated monocyte counts in IPF have been associated with worse outcomes (30, 31). Growing data also shows monocyte-derived cells in lungs display discrete profibrotic phenotypes characterized by the expression of markers of alternative macrophage activation (32). In addition, macrophages are activated by activators such as IFN-γ, IL-10, or IL-3, acquiring profibrotic phenotype (33). Even more, macrophages can be polarized to M1 or M2 by these chemokines and release TGF-β and IL-10 to regulate endothelial cell proliferation, fibroblast activation, angiogenesis, and extracellular matrix (ECM) deposition to facilitate fibrosis formation (34, 35). Few T cells are in the fibrotic lung compared to the healthy lung (36). Above all, we believe that cluster 2 IPF patients are more likely to have worse outcomes, but more studies are needed. GO enrichment showed that the Cluster 2 IPF group was enriched in the ubiquitin ligase complex, ubiquitin-mediated proteolysis, tRNA methylation, monocyte aggregation, nucleotide sugar metabolic process, cell-cell adhesion *via* plasma membrane adhesion molecules, circulatory system development, myotube differentiation, and synaptic membrane, among others. KEGG pathway enrichment showed that Cluster 2 IPF was enriched in aminoacyl tRNA biosynthesis, RNA polymerase, calcium signaling pathway, and other pathways.

The performance of 4 selected machine-learning models (RF, SVM, GLM, and XGB) was compared and selected based on the high predictive efficacy in the testing cohort. Results showed that the XGB-based machine-learning model had the best performance in predicting the IPF. We then selected 4 critical genes (XKR6, MLLT3, CD40LG, and HK3) to construct a 4-gene-based XGBand nomogram models. The constructed 4-gene-based XGB model could accurately predict IPF, validated in other external datasets (AUC = 0.700), which provides new insights into the diagnosis of IPF. The nomogram was established for the diagnosis of IPF, exhibiting effective predictive efficacy with possible clinical application. Next, we analyzed the correlations between the clinical characteristics of IPF and 4 critical genes. DLCO was used to evaluate the diffusing capacity of the lung for carbon monoxide and aiding in IPF diagnosis. Our result revealed that only DLCO strongly correlated with the selected 4 genes. Additionally, an increasing number of studies have confirmed that TGF-β1 is a fundamental pathological mechanism, which contributes to the progression of IPF by promoting the transformation of fibroblast into myofibroblast, epithelial cells into mesenchymal cells, the production of collagen, filamentous actin, and α-SMA (37). Therefore, we performed a correlation analysis between these 4 predictor genes and TGF-β in two databases. The results suggested that HK3 was positively associated with TGF-β1, while the other 3 predictor genes were negatively correlated with TGF-β1 levels. Overall, the 4-gene-based XGB model is a satisfactory indicator of the diagnosis of IPF.

We also constructed a gene-gene network and performed Go and KEGG analyses of similar genes related to the 4 critical genes. GO analysis of CD40LG showed that tumor necrosis and NF-κB were primarily enriched. Many studies have demonstrated that the tumor necrosis factor is primarily produced by macrophages and monocytes linked to a number of pulmonary inflammatory diseases, including IPF (38, 39). It also has been widely reported that NF-κB is one of the essential pathways in the progression of IPF, and blockade of NF-κB prevented lung fibroblast-mediated IL-6, IL-8, and CXCL6 cytokine secretion as well as accumulation of profibrotic factors (40). Meanwhile, regulation of the immune and tumor necrosis factor-mediated signaling pathways are enriched in KEGG. Therefore, CD40LG may correlated with the progression of IPF and the immune system. HK3, one of the 4 critical genes, is a protein-coding gene related to the glycolysis pathway. It has been observed that glycolysis reprogramming drives fibroblast activation when macrophages direct the metabolic fate of adjacent cells, implying that HK3 may be influenced in the development of IPF (41). MLLT3, as a critical gene, acts upstream of or within the negative or positive regulation of the canonical Wnt pathway, which has been reported to be associated with lung fibroblast activation, differentiation, and dysregulation of repairing processes (42). Although the correlation between IPF and MLLT3 has not been reported, we believe that MLLT3 may play a role in regulating the Wnt signaling pathway to participate in the progression of IPF. In addition, the correlation of XKR6 with IPF has not been reported. However, the mechanism of the 4 critical genes in regulating IPF progression needs more studies.

This study has some limitations. Firstly, more IPF samples are needed to demonstrate the correlation between CRGs and IPF disease or immune cells infiltration. Secondly, it is necessary to do more experiments to clarify the regulation and mechanism of the 4 critical genes identified and CRGs in the progression of IPF. Lastly, more clinical features are required to confirm the validity of the predictive model.

## 5 Conclusions

In conclusion, our study clarified that CRGs might play a role in IPF progression. We also showed the correlation between CRGs and immune cell infiltration, and elucidated the significance of immune heterogeneity in IPF patients with distinct cuproptosis clusters. The prognostic model based on the 4 critical genes may allow a new way to predict the prognosis of IPF.

## Data availability statement

The original contributions presented in the study are included in the article/supplementary material. Further inquiries can be directed to the corresponding authors.

## Author contributions

XS and ZP wrote and revised the manuscript. YZ, WC and JD collected the original data and visualized the final results. XS provided the funding. TC and RL supervised the study. The final manuscript was read and approved by all authors. All authors contributed to the article and approved the submitted version.
